# Arthroscopic Suture Bridge Combined All-Inside Fixation for Delaminated Rotator Cuff Tears

**DOI:** 10.1016/j.eats.2024.103276

**Published:** 2024-11-14

**Authors:** Hong Qian, Xiaojiang Yang, Zhongyang Lv, Shao Yu, Jingwei Lu, Jichun Liu, Ninrong Bao

**Affiliations:** a Department of Orthopedics, Jinling Hospital, School of Medicine, Nanjing University, Nanjing, China; b Department of Education and Support, Army Engineering University of PLA, Nanjing, China; c Department of Sports Medicine, Tongji Hospital, School of Medicine, Tongji University, Shanghai, China; d Department of Orthopedics, Nanchang Hospital, Nanchang, China

## Abstract

The anatomic repair of delaminated rotator cuff tears presents a surgical challenge, necessitating innovative solutions for optimal outcomes. This Technical Note describes the arthroscopic suture bridge combined with all-inside fixation, a novel approach tailored for small yet deep-layer dominant delaminated tears. The method involves addressing the upper-layer tear beneath the acromion and employing all-inside suturing in the glenohumeral joint for the lower-layer tear, decoupling the outer tear size from limiting the treatment of the deeper tear. Accurate tear identification and preoperative planning are critical for success. This technique offers advantages in overcoming the outer tear’s impact on lower tear repair, potentially reducing patient costs. Proficiency in tear identification and surgical planning is essential for successful execution. Considerations include reserving sutures as a precaution and inapplicability to posteriorly located delaminated tears. This approach provides a valuable contribution to arthroscopic techniques, especially for smaller delaminated tears.

The primary objective of arthroscopic rotator cuff repair is to meticulously reattach the rotator cuff tendons to their native footprint, ensuring an anatomically precise, tension-free union.^1^, ^2^, ^3^, ^4^ This aims to restore a natural bone-tendon junction, optimizing physiological function. This imperative holds especially true for cases of delaminated rotator cuff tears. Failing to address rotator cuff delamination during repair has been associated with lower healing rates postoperatively.^5^^,^^6^

Delaminated tears are characterized as a form of degeneration within the tendon, involving a horizontal split in the tendon substance with associated vascular changes.^7^ The recognition of delamination in rotator cuff tears is gaining prominence in the literature, suggesting that it may occur more frequently than previously appreciated.^8^^,^^9^ Clinically, 2 major layers—the superficial bursal and deeper articular sides—are discernible. In instances of delamination, the deeper layer often exhibits greater retraction compared to the superficial layer.^10^ While the exact cause of delamination remains uncertain, factors such as local ischemia and uneven stress distribution between the layers of the cuff have been postulated to contribute to its development.^11^

Addressing delamination properly during surgical intervention is paramount, as failure to do so can adversely affect the outcome of rotator cuff repairs. Understanding the distinctive characteristics of these layers and recognizing the presence of delamination are crucial for achieving successful surgical outcomes and restoring optimal shoulder function. The restoration of both the articular and bursal layers onto the footprint, taking into account the retraction pattern and repair tension of each layer, is believed to be of utmost importance in preserving the structural integrity following cuff repair.

Delaminated rotator cuff tears present technical challenges for achieving anatomic repair and may lead to less favorable outcomes. Several studies have indicated that the presence of delaminated tears negatively impacts functional and radiologic results following rotator cuff repair.^5^^,^^6^ To address this, various techniques involving separate suturing of each laminated layer have been introduced.^12^, ^13^, ^14^ However, these methods are primarily designed for larger or massive rotator cuff injuries, and we believe that the existing methods are inadequate for addressing small but deep-layer dominant delaminated tears.

The objective of this report is to elucidate our method—arthroscopic suture bridge combined with all-inside fixation—for anatomically repairing both layers of delaminated rotator cuff tears individually, specifically tailored to address the challenge posed by these smaller but deep-layer dominant tears.

## Surgical Technique

After induction of general anesthesia, the patient is placed in the lateral decubitus position using a beanbag to ensure stability. To minimize the risk of neural injury, an axillary roll is positioned, and proper padding is applied to the lower leg. An examination under anesthesia is then conducted to evaluate stiffness. Subsequently, the operative extremity is suspended using approximately 10 pounds of balanced traction.

**Fig 1:**
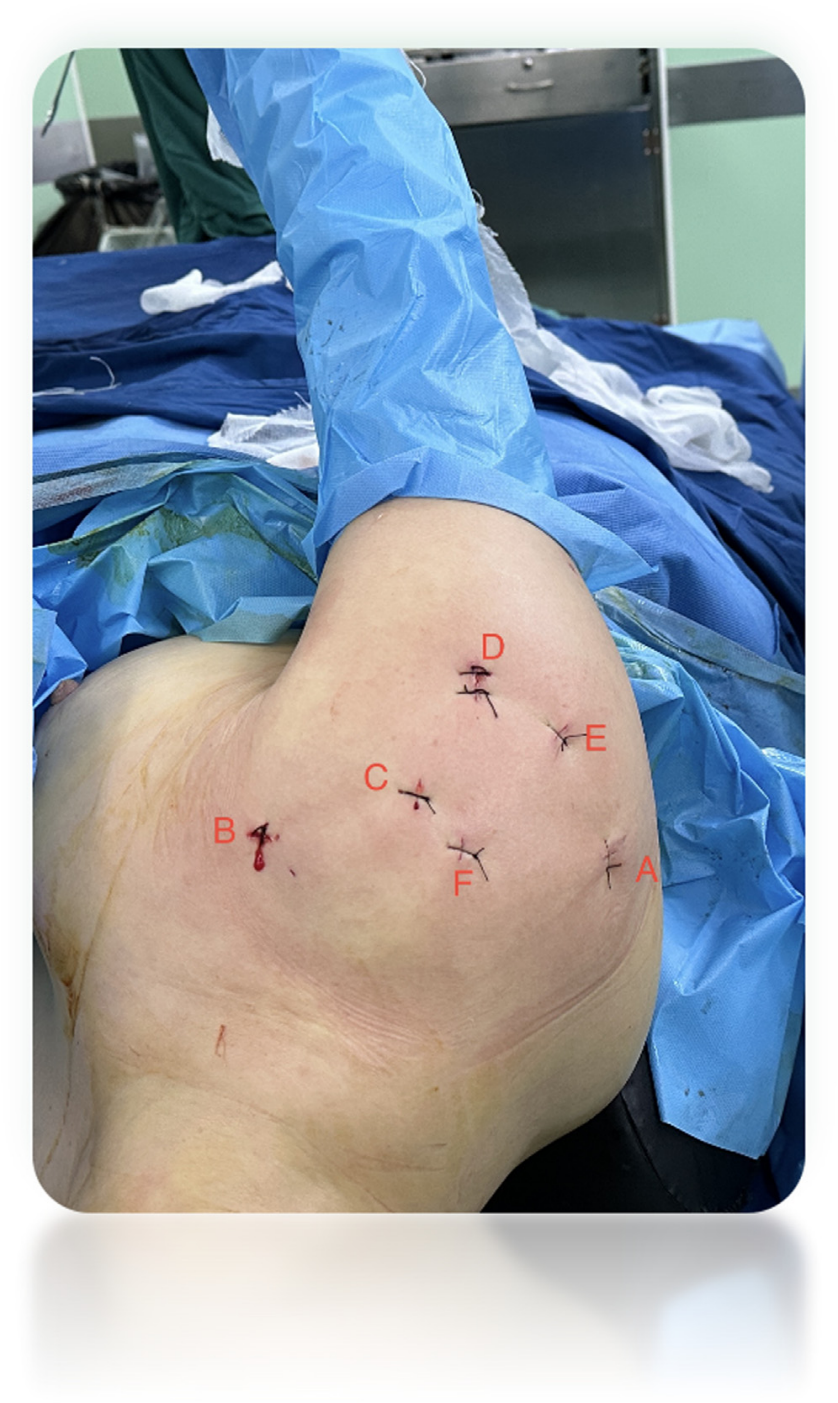
Preoperative photograph showing the patient in the lateral decubitus position with the right arm in traction using an axillary support. The portals are marked on the skin: posterior (A), anterior (B), anterosuperior (C), lateral (D), posterior-lateral (E), and anterior-lateral (F).

A standard posterior viewing portal is established, followed by the creation of an anterior portal in the rotator interval (Fig 1). A comprehensive diagnostic arthroscopy is performed to explore potential intra-articular pathologies, including adhesions in both the anterior and posterior capsules, tears in the subscapularis tendon, and issues related to the biceps tendon. If deemed necessary, corrective procedures such as subscapularis tendon repair and biceps tenotomy or tenodesis are performed at this stage.

**Fig 2:**
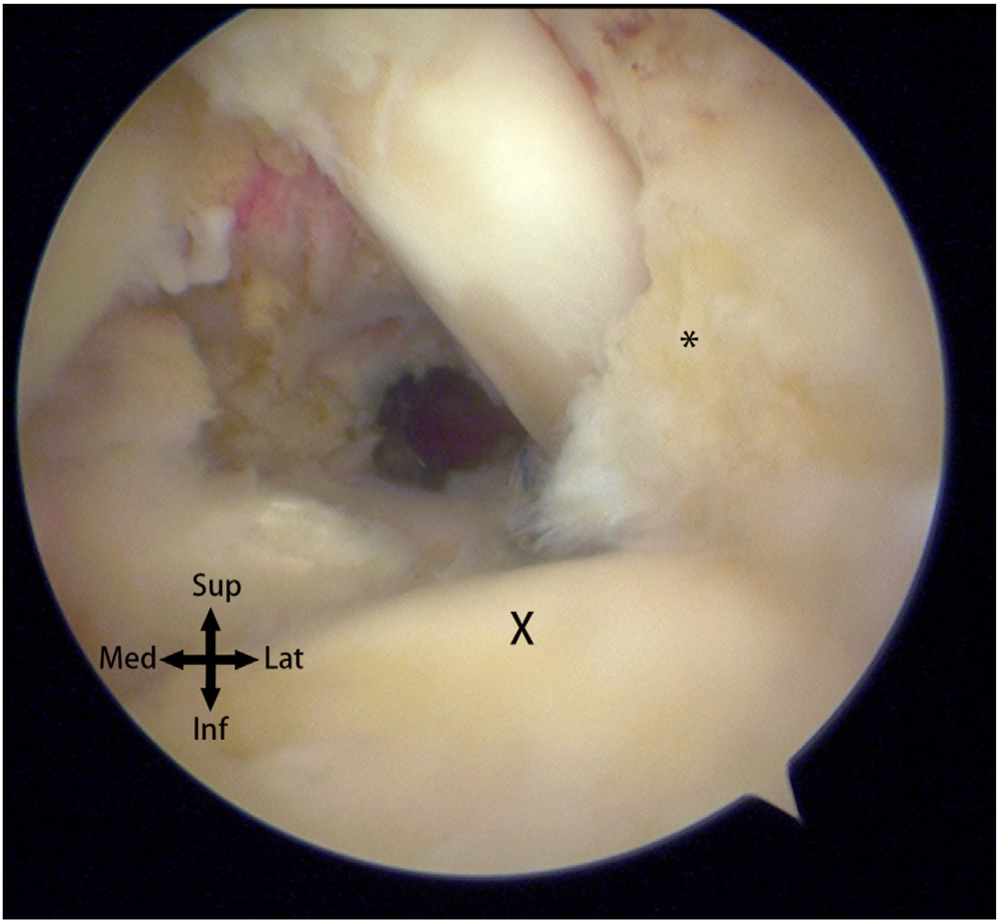
The view from the posterior portal in the lateral decubitus position. X indicates the articular surface of the humeral head, while the asterisk (∗) indicates the torn edge of the undersurface. However, it is challenging to assess the extent of retraction of the undersurface tear toward the medial side.

**Fig 3:**
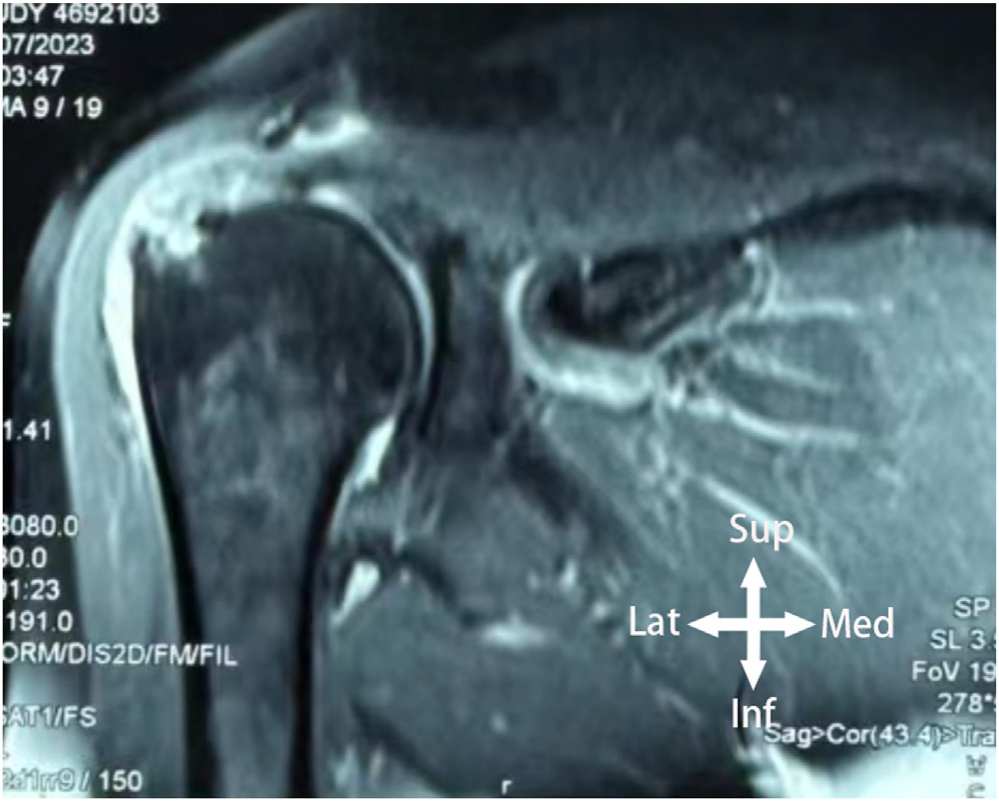
The patient’s magnetic resonance imaging reveals a rotator cuff injury with delaminated tears on the superior and inferior surfaces of the supraspinatus muscle.

When encountering delaminated tears of the rotator cuff, in cases of small but deep-layer dominant delaminated tears, determining the extent of articular surface involvement through the posterior portal alone can be challenging (Fig 2). Therefore, after completing essential procedures in the glenohumeral joint, the scope is transitioned from the glenohumeral joint to the subacromial space. Accurate assessment of these tears requires the surgeon’s experience, incorporating preoperative magnetic resonance imaging (Fig 3) and intraoperative observation of incongruent retraction distances between the articular surface and the bursal side. This becomes a crucial aspect of the procedure.

**Fig 4:**
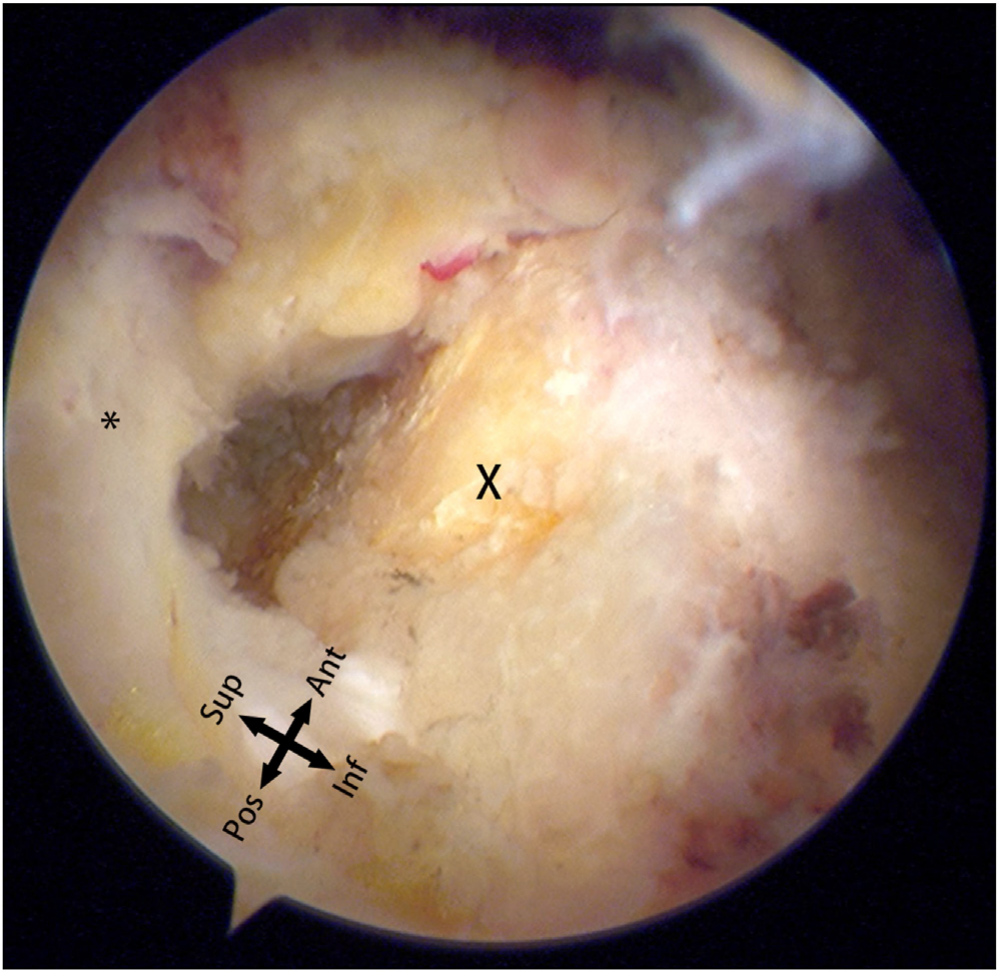
The defect (∗) and footprint area (X) on the superior surface of the supraspinatus muscle observed from the posterior-lateral approach into the subacromial space.

**Fig 5:**
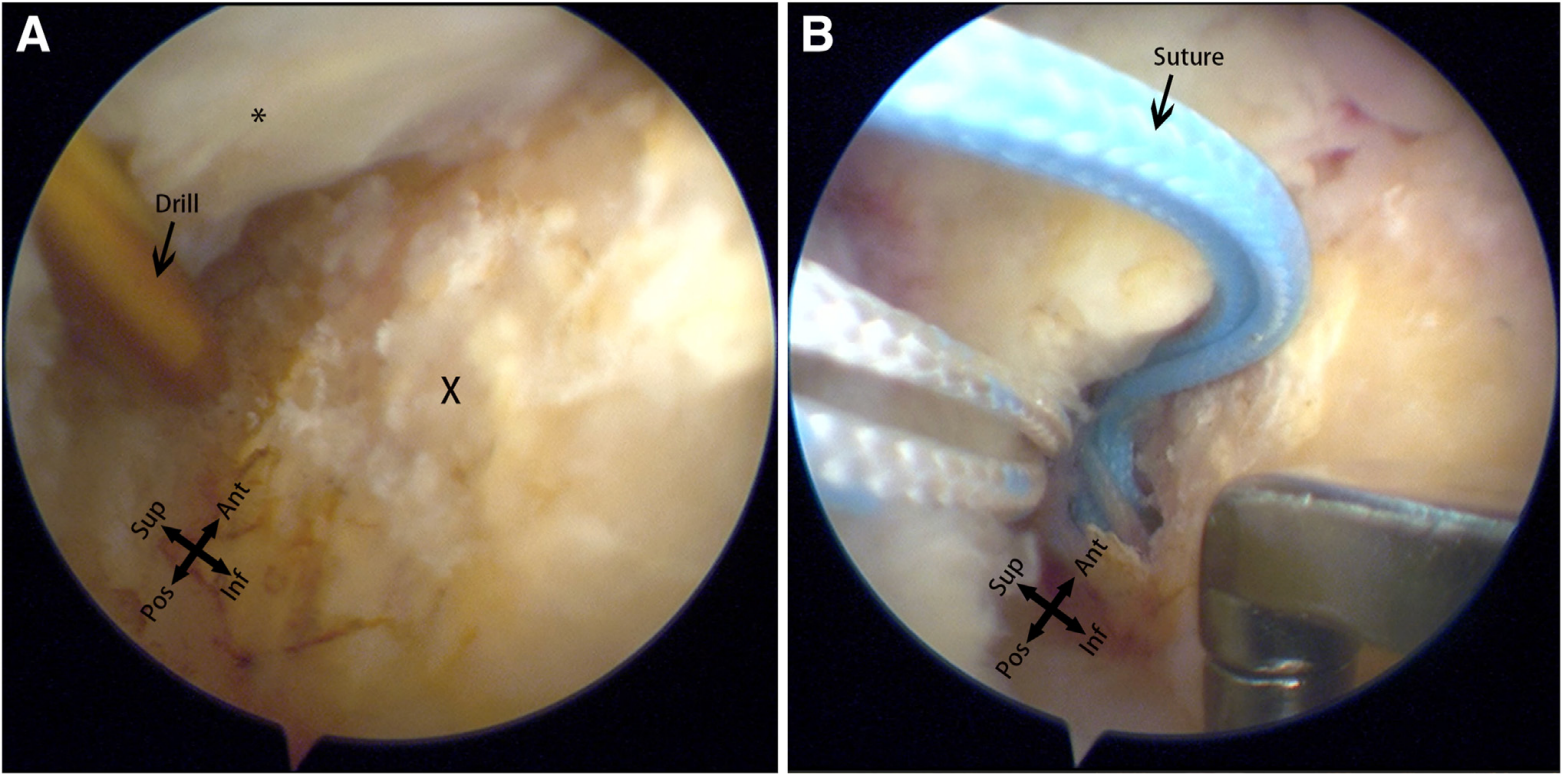
(A) Insert a drill through the anterior-lateral portal, positioning it snugly against the lateral edge of the cartilage. (B) After the insertion of the anchor, a set of sutures is passed through the defect in the supraspinatus muscle using graspers through the lateral portal, reaching into the glenohumeral joint.

**Fig 6:**
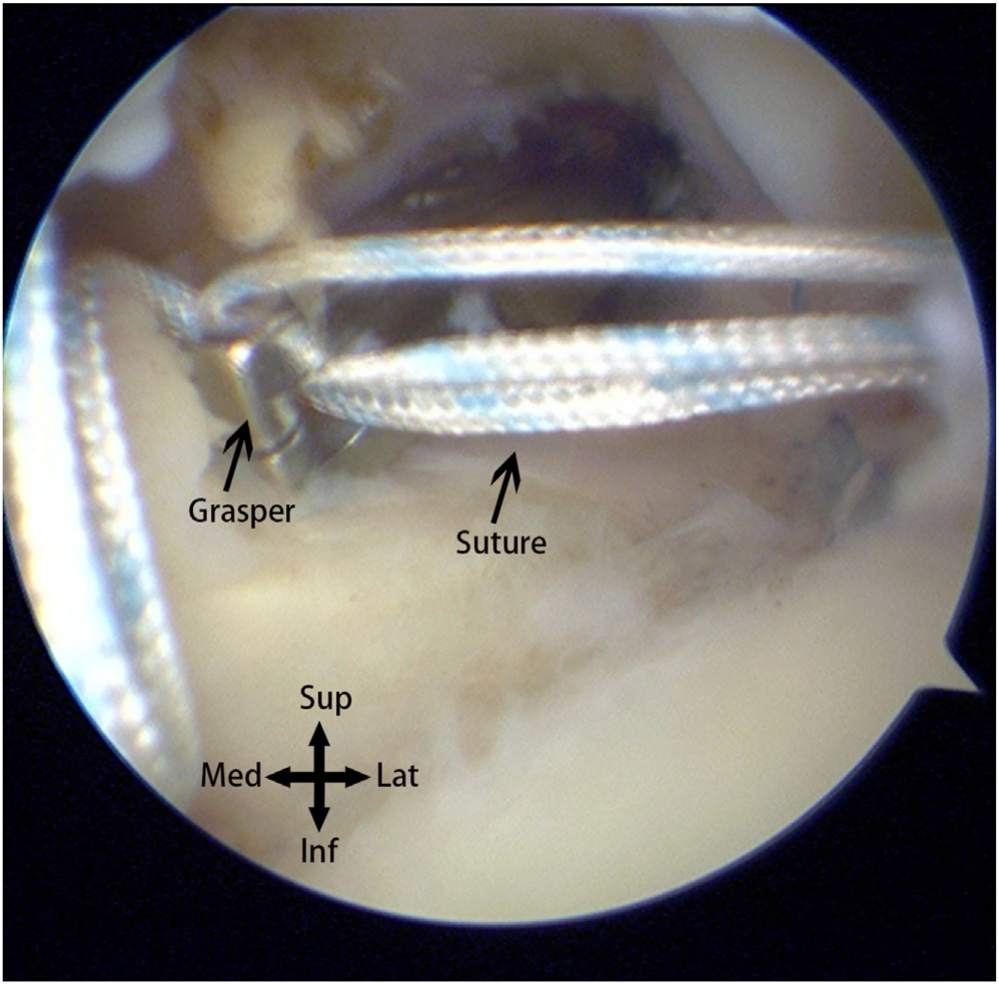
Switching the camera to the posterior portal to access the glenohumeral joint, using graspers to retrieve the preplaced sutures through the anterior portal to the outside of the body for further use.

**Fig 7:**
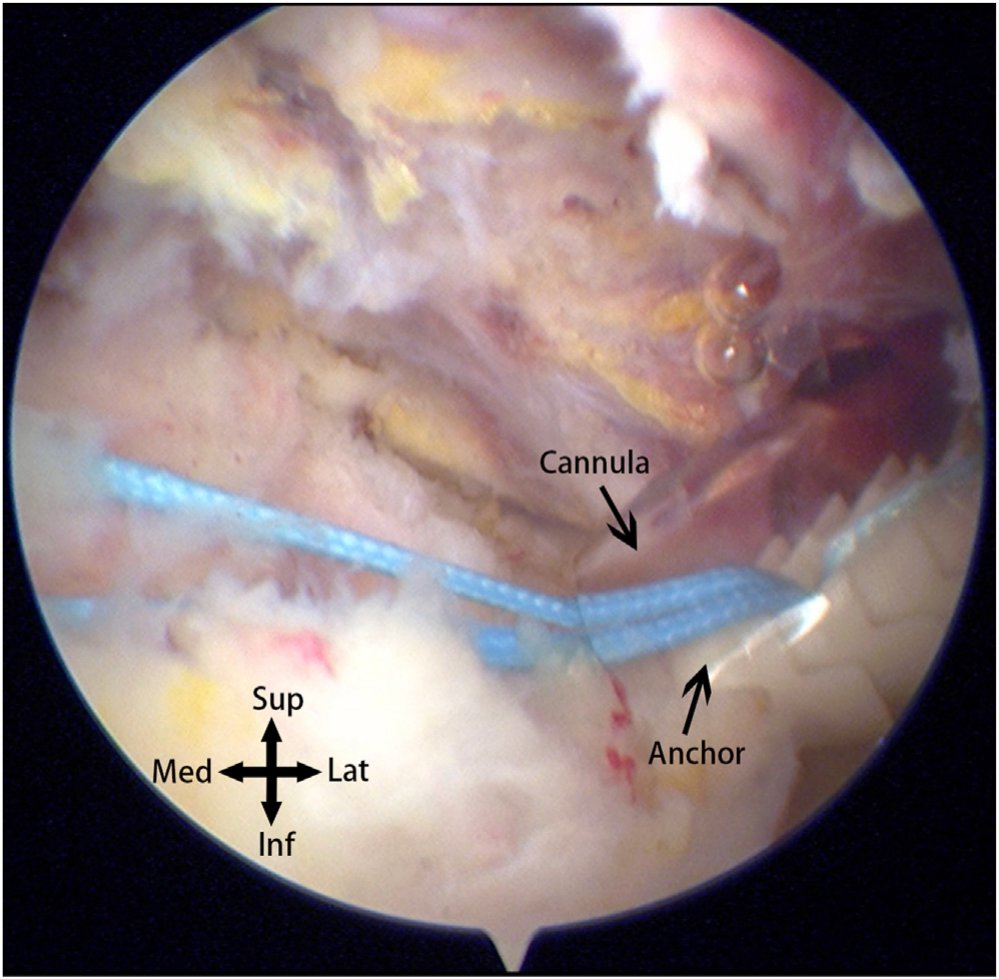
The camera enters the subacromial space through the posterior portal, and a cannula is inserted through the lateral portal. The suture bridge technique is then employed to repair the damaged superior surface of the supraspinatus muscle.

Moving to the subacromial space, observation reveals a relatively small, anteriorly located defect on the upper surface (Fig 4). Based on preoperative magnetic resonance imaging and previous surgical experience, this is determined to be a case of small-sized delaminated but deep-layer dominant tears. To facilitate observation and cleaning, a posterior lateral portal is established, and the viewing portal is switched from the posterior to the posterior lateral portal. Using a plasma wand for cleaning, a burr is employed through anterior lateral portal 1 for bone freshening. Close to the lateral edge of the acromion, an anterior lateral portal is established. A 4.5-mm dual-threaded suture anchor (Helico; Smith & Nephew) is inserted through this portal (Fig 5A), and a suture grasper through the lateral portal is used to insert threads through the upper surface fissure into the glenohumeral joint (Fig 5B). The camera is switched to the posterior portal for observation, and a suture retriever through anterior lateral portal 1 pulls out threads for later use (Fig 6). The camera is switched to the subacromial space, and using a suture hook, the suture is passed through the upper surface of the supraspinatus tendon twice. Through the lateral portal, a 7-mm working cannula is inserted, and a 4.5-mm outside-in suture anchor (Smith & Nephew) is inserted (Fig 7). The suture bridge technique repairs the upper surface damage.

Switching the camera to enter the glenohumeral joint space through the posterior portal, the upper surface of the delaminated tear is repaired. Despite the medial position of the suture hook needle during upper surface stitching, due to significant retraction of the lower surface tear and lack of support, the suture only repairs the upper surface. Without accurate identification of concealed delaminated tears, using only the suture bridge technique for repair in the subacromial space, without re-entering the glenohumeral joint for inspection, may overlook damage to the lower surface. Even if damage to the lower surface is discovered at this point, repairing it without a reserved set of sutures in the glenohumeral joint becomes tricky.

**Fig 8:**
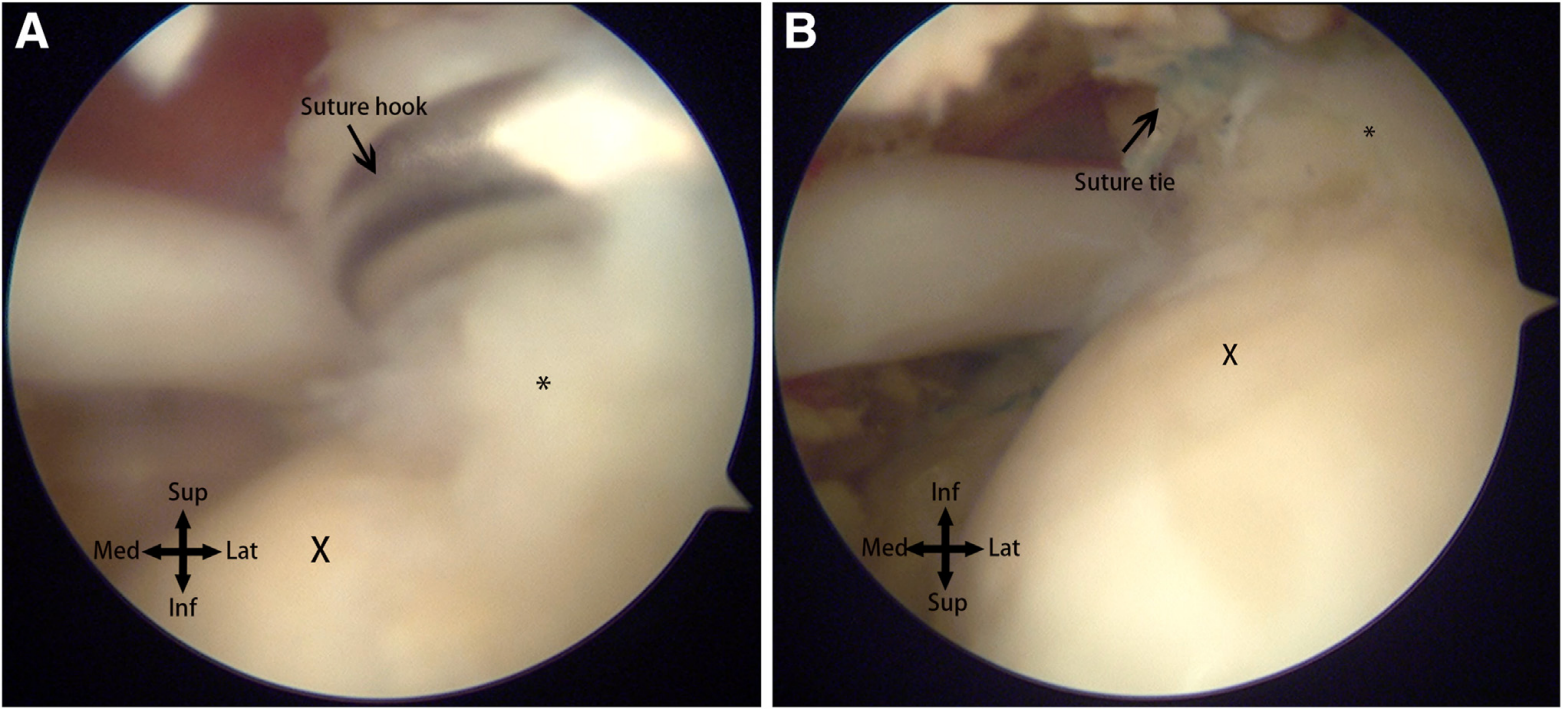
(A) The camera re-enters the glenohumeral joint space through the posterior portal. Utilizing a suture passer through the anterior portal, the damaged undersurface of the supraspinatus muscle is repaired using an all-inside suturing technique. (B) The repair of the undersurface of the supraspinatus muscle is complete. The suture knots indicated by the arrow are relatively small. The torn edge (∗) of the undersurface returns to its footprint area, while the exposed area of the humeral head cartilage (X) noticeably increases.

With preoperative planning and surgical experience, and given sensitivity and accuracy in judging these types of delaminated tears, this is a case of a simple supraspinatus undersurface injury. The reserved set of sutures is used, with a suture hook using an all-inside suture technique to repair the undersurface damage. Passing the suture twice and using a mattress suture technique increases the transverse area of the tendon held by the suture (Fig 8A). After passing the suture, a suture retriever from anterior lateral portal 1 is used to enter the glenohumeral joint space, simultaneously grabbing the 2 threads for tying using a pusher. Usually, 5 knots are tied, and excess suture is cut using a suture cutter (Fig 8A). Pearls and pitfalls as well as advantages and disadvantages of the technique are described in Tables 1 and 2. The surgical technique is demonstrated in Video 1.

**Table 1:** Surgical Pearls and Pitfalls

**Pearls**
The approach involves addressing the upper-layer tear below the acromion and utilizing an all-inside suture technique for the lower-layer tear in the glenohumeral joint. This ensures that the extent of damage in the outer layer no longer constrains the management of the lower-layer tear. Performing layered sutures both above and below allows for matching tension on the upper and lower surfaces, facilitating a more effective anatomic repair of the footprint area. Intraoperative discernment of proper layering and extensive surgical experience are crucial for employing this method in the repair of rotator cuff injuries. Reserving an additional set of sutures when uncertain can serve as a precautionary measure.
**Pitfalls**
This technique is not suitable for posteriorly located delaminated tears.
Switching between operating spaces during surgery requires skilled surgical techniques and a good sense of touch.
During the surgery, having keen judgment is crucial for implementing this procedure.
The surgeon should possess proficient skills in the use of the all-inside suture technique and demonstrate effective suture management.

**Table 2:** Advantages and Disadvantages

Advantages	Disadvantages
• Anatomic restoration: The technique facilitates the anatomic restoration of both articular and bursal layers onto the footprint, contributing to the overall structural integrity after cuff repair. • Addressing delamination: Specifically designed for delaminated rotator cuff tears, it effectively addresses the challenge posed by the retracted articular layer, providing a tailored solution for complex tear patterns. • Tension matching: The layered approach enables the matching of tension between the articular and bursal layers, optimizing the repair for improved biomechanics and reducing the risk of structural failure. • Enhanced healing process: By avoiding knots between layers and utilizing self-cinching stitches, the technique promotes increased interlayer contact between fibers, potentially enhancing the healing process.	• This technique is not suitable for posteriorly located delaminated tears. • Surgeons adopting this technique may face a learning curve due to its complexity and specific steps. Adequate training and practice are essential for successful implementation.

## Rehabilitation

A postoperative rotator cuff repair protocol is implemented, involving placing the patient in an arm sling with an abduction pillow. Immediate engagement in tabletop activities without the sling is permitted after surgery. Core and periscapular exercises commence immediately as well. The arm sling is consistently worn for the initial 4 weeks postsurgery. Subsequently, active assistive range of motion and stick exercises are initiated between postoperative weeks 4 and 5. Strength-building exercises and active resistance training are encouraged starting at the 3-month mark. Full resumption of activities, including sports and heavy labor, is permitted after 6 months.

## Discussion

Previous studies have consistently highlighted that the presence of delamination during arthroscopic rotator cuff repair is a negative prognostic factor.^5^^,^^6^ Recognizing the potential impact of delamination on structural integrity after cuff repair, achieving the anatomic restoration of both the articular and bursal layers onto the footprint becomes crucial.^2^ Delamination often results in greater retraction of the articular layer compared to the bursal layer,^10^ demanding intricate repair techniques. In this Technical Note, we present and discuss our preferred method—arthroscopic suture bridge combined with all-inside fixation—for effectively addressing delaminated rotator cuff tears.

Although many have proposed various arthroscopic methods for treating delaminated rotator cuff tears, we believe existing approaches are insufficient for handling all types of these tears. Commonalities among previous methods include targeting large or massive delaminated tears and conducting cuff repair under the acromion. The main differences lie in the fixation methods for addressing superficial articular layer tears. A wide variety of modifications have been proposed to these techniques, including the rip-stop suture bridge,^15^ the tension-band suture technique,^16^ the Roman bridge technique,^17^ the hybrid technique,^18^ and a technique combining a modified SpeedBridge (Arthrex) with the double pulley technique.^19^ When facing medium or small delaminated tears, especially those with a small bursal layer opening and significant retraction of the articular layer, as highlighted in the initial description of small but deep-layer dominant delaminated tears, it is challenging to observe, grasp, suture, and pass threads through the inner aspect of the tear without artificially enlarging the subacromial space via the acromial portal. These steps require the synchronous operation of observation, grasping, and suturing through 3 separate channels, which is difficult with a limited acromial portal size.

Our techniques involve layered suturing of both upper and lower surfaces, but with a significant difference. We suture the upper surface in the subacromial space, while the lower surface is repaired by returning to the glenohumeral joint space using an all-inside suture technique. This approach allows us to suture the lower surface without being influenced by the small opening in the upper surface. Moreover, it enables effective anatomic restoration of the footprint’s upper and lower surfaces through layered suturing. Our suturing method has the potential for reducing the overall cost for patients.

The essential qualities for surgeons are accurate identification of delaminated tears and effective intraoperative planning. First, delaminated tears are more prevalent than previously thought. Admitting that, due to our lack of clinical experience and insufficient understanding of delaminated tears, many were treated as common rotator cuff tears in the past. Accurate identification of the tear type and preoperative planning of the suturing strategy, along with prepositioning of glenohumeral joint sutures, are vital for the systematic use of this technique for layered repair of small but deep-layer dominant delaminated tears.

While we need to switch observation channels, preposition sutures, and perform all-inside suturing, we found through clinical practice that proficiency in this technique, coupled with good surgical planning, adds only about 10 minutes to the overall surgery time compared to the standard suture bridge technique. Additionally, we recommend reserving a set of sutures to the glenohumeral joint space as a precautionary measure, especially when uncertain about whether the rotator cuff is delaminated. This serves as both a good surgical habit and a remedial measure.

## Disclosures

All authors (H.Q., X.Y., Z.L., S.Y., J. Lu, J. Liu, N.B.) declare that they have no known competing financial interests or personal relationships that could have appeared to influence the work reported in this paper.

## Supplementary Data

Video 1 In this video, we present our technique for arthroscopic suture bridge combined all-inside fixation for delaminated rotator cuff tears. We prefer performing this procedure with the patient in the lateral decubitus position. Viewing from the posterior portal, an anterior portal in the rotator interval is created. A comprehensive diagnostic arthroscopy is performed to explore potential intra-articular pathologies, and the scope is then transitioned from the glenohumeral joint to the subacromial space. After performing subacromial synovial membrane cleaning and hemostasis, we observe significant proliferation on the undersurface of the acromion, with a defect on the upper surface of the supraspinatus muscle. To better visualize the footprint area, we switch the viewing portal from the posterior to the posterior-lateral portal. A 4.5-mm dual-threaded suture anchor (Helico; Smith & Nephew) is inserted, and a suture grasper through the lateral portal is used to insert threads through the upper surface fissure into the glenohumeral joint. The camera is switched to the posterior portal for observation, and a suture grasper through anterior portal 1 pulls out threads for later use. The camera is switched to the subacromial space, and using a suture hook, the suture is passed through the upper surface of the supraspinatus tendon twice. Through the lateral portal, a 7-mm working cannula is inserted, and a 4.5-mm outside-in suture anchor (Smith & Nephew) is inserted. The suture bridge technique repairs the upper surface damage. Switching the camera to enter the glenohumeral joint space through the posterior portal, due to significant retraction of the lower surface tear and lack of support, the suture only repairs the upper surface. The reserved set of sutures is used, with a suture hook using an all-inside suture technique to repair the undersurface damage. At this point, the footprint is anatomically repaired. After the completion of the procedure, all portals are meticulously closed with interrupted absorbable sutures, and the patient’s arm is immobilized in a shoulder abduction sling.
